# Different age groups present different clinics in anaphylaxis with children: one size does not fit all children

**DOI:** 10.55730/1300-0144.5609

**Published:** 2023-02-13

**Authors:** Nevzat BAŞKAYA, Ayşegül ERTUĞRUL, Saliha ESENBOĞA, Serap ÖZMEN

**Affiliations:** Department of Pediatrics, Division of Allergy and Immunology, University of Health Sciences, Dr Sami Ulus Maternity and Children Research and Training Hospital, Ankara, Turkey

**Keywords:** Allergy, anaphylaxis, children

## Abstract

**Background/aim:**

Childhood anaphylaxis presents with a heterogeneous clinic. Elicitors and epidemiologic factors associated with anaphylaxis differ with age, geographic location and lifestyle. This study aimed to determine the clinical features and age-specific patterns of childhood anaphylaxis in a single referral center in Turkey.

**Materials and methods:**

We conducted a retrospective study of anaphylaxis in children aged between 0 and 18 years of age, attending an allergy department in a children’s hospital.

**Results:**

A total of 95 children diagnosed with anaphylaxis were analyzed. Among all, 35.8% of the first anaphylaxis episodes occurred in infancy and 57.9% in preschool age. Foods were the most common culprits (57.9%) and followed by drugs (15.8%). Patients with food-induced anaphylaxis were younger in age (p < 0.001). Food-related anaphylaxis was most common with cow’s milk (36.4%) and followed by tree nuts (20%). Cow’s milk played a significant role as a trigger in infancy, and tree nuts as a trigger in preschoolers and school-age children. Mucocutaneous manifestations were almost universally present (94.7%), followed by respiratory compromise (56.8%), with gastrointestinal (55.8%), cardiovascular (9.5%), and neurologic (4.2%) symptoms being less common. Respiratory and cardiovascular system-related symptoms were found more frequently in school-age children (p = 0.02 and p = 0.014, respectively). The severity of anaphylaxis was higher in school-age children (p = 0.015).

**Conclusion:**

Findings reveal that children diagnosed with anaphylaxis differ in terms of etiological and clinical findings according to age groups. This difference shows the dynamically changing clinic of anaphylaxis over time and the importance of evaluating childhood anaphylaxis according to age groups.

## 1. Introduction

Anaphylaxis is a severe, systemic and potentially life-threatening hypersensitivity reaction characterized by being rapid in onset [1]. The incidence of anaphylaxis appears to be increasing worldwide in recent decades, but most studies have focused on adults [2]. Anaphylaxis is becoming more common, according to a multicenter research conducted in Turkey [3]. Clinical manifestations of anaphylaxis in children differ from those in adults, implying clinical heterogeneity in pediatric anaphylaxis across age groups [2,4]. Food is the most common cause of anaphylaxis in children but elicitors and epidemiologic factors associated with anaphylaxis differ with age, geographic location, culture and lifestyle [1,4].

In the current study, we aimed to identify childhood anaphylaxis in a tertiary care referral center to figure out our patients’ demographics, main elicitors and symptoms of anaphylaxis, and management of patients. We determined the age-specific patterns of childhood anaphylaxis.

## 2. Materials and methods

### 2.1. Patients and data collection

Patients who were followed in a tertiary care children’s hospital for anaphylaxis between January 2018 and August 2020 were identified for screening by extracting ICD-10 anaphylaxis codes (T78.0 Anaphylactic shock due to adverse food reaction, T78.2 Anaphylactic shock, unspecified, T80.5 Anaphylactic reaction due to serum, T88. 6 Anaphylactic reactions due to adverse effect of correct drug or medicament properly administered) from the electronic medical file system retrospectively. The medical files were reviewed in detail by a researcher (N.B.) for anaphylaxis. Patients, aged 0–18 years, only with a diagnosis of anaphylaxis according to the clinical criteria of the World Allergy Organization (WAO), regardless of the underlying cause were included [1]. Anaphylaxis was described with two criteria; a. Acute onset of illness involving skin and/or mucocutaneous manifestations and at least one of the following organ systems: the respiratory, circulatory, and gastrointestinal systems, b. Acute onset of hypotension/bronchospasm/laryngeal involvement after exposure to a known or highly probable allergen for that patient, even in the absence of typical skin involvement. Patients who did not match the WAO definition or patients with missing data in medical files were excluded from the study.

Demographics (age at episode, sex), symptomatology (type, onset, timing, location, and recurrence), severity based on the Ring and Messner classification system, diagnostic investigations (complete blood count, total Ig E, tryptase, specific IgE, skin prick and/or intradermal test, allergen provocation test), eliciting triggers, exacerbating factors, personal and family history of allergic disease, concomitant diseases, emergency treatment were recorded on a standard form [5].

The triggering factor was identified by allergen skin testing and/or allergen provocation test that correlated with clinical history. Idiopathic anaphylaxis (IA) is considered when no specific trigger can be identified after an appropriate evaluation.

### 2.2. Statistical analysis

SPSS (Statistical Package for Social Sciences) program 22.0 (IBM Corp., Armonk, NY, USA) was used for statistical analysis. The data were analyzed with descriptive statistics such as frequency and percentage distribution. Where appropriate, categorical comparisons were analyzed using Pearson’s chi-square or Fisher’s exact probability test. Comparison of continuous variables was made using Student’s t-test or Mann-Whitney U test. Statistical significance was regarded as p < 0.05.

This study was carried out by the principles of the Declaration of Helsinki, with the ethical approval (numbered 93471371-514.10) obtained from the Ankara Training and Research Hospital Ethics Committee.

## 3. Results

### 3.1. Demographics and symptomatology

We evaluated the medical records of 95 of the 9088 patients who presented to the pediatric allergy outpatient clinic between 2018 and 2020 and were diagnosed with anaphylaxis. Fifty-eight were male (61.1%), with a median age of 77 months [interquartile range (IQR): 29–133 months]. Forty-nine patients (51.6%) were considered to have an accompanying allergic disease. Clinical and demographic features are shown in Table 1.

Mucocutaneous manifestations were almost universally present (94.7%), followed by respiratory compromise (56.8%), with gastrointestinal (55.8%), cardiovascular (9.5%), and neurologic (4.2%) symptoms being less common. None of the instances recorded was classified as mild, with 81 cases (85.3%) being grade 2, and the remainder (14.7%) being grade 3. Information on the time interval between the exposure of the trigger and the onset of anaphylaxis symptoms was available in seventy-eight cases. Trigger exposure-symptom onset time was less than 1 h in seventy patients (81.4%). It was between 1 h and 4 h in 6 patients, and longer than 4 h in 1 patient with red meat anaphylaxis. Biphasic reactions occurred in three patients (3.2%). There were no fatalities during the data collection period. One-tenth (12.6%) of all events occurred in a healthcare setting, while 72.6% of events happened at home. Twelve episodes of anaphylaxis occurred in the healthcare setting, nine of which were caused by drugs, one by perioperative latex exposure, one by vaccine administration, and one by skin prick testing with a fish allergen. Demographic characteristics of the patients and symptoms of anaphylaxis are compared according to different age groups, shown in Table 2. Respiratory and cardiovascular system-related symptoms were found more frequently in school-age children (p = 0.02 and p = 0.04, respectively). Gastrointestinal system symptoms were observed more frequently in preschool-age children (p = 0.02).

### 3.2. Diagnosis and testing

Skin prick testing and specific IgE assays were the mainstays of confirmation of diagnosis and were used in 87.2% and 65.4% of reactions, respectively. The median (IQR) total IgE level and absolute blood eosinophil count were 60 IU/mL (19–104) and 170 cells/L (100–280), respectively. There were no significant differences in the absolute blood eosinophil count and total IgE level regarding patients’ age groups, anaphylaxis severity or anaphylaxis triggers (p > 0.05). During anaphylaxis, tryptase levels were requested in 29.4% of episodes. The median (IQR) tryptase level was 5.1 μg/L (2.8–7.9) with a maximum level of 30.4 μg/L. The basic tryptase levels were evaluated in 27.4% of the patients. The median (IQR) tryptase level was 4.19 μg/L (2.9–5.6) with a maximum level of 9.33 μg/L. There were no significant differences in the tryptase levels regarding patients’ age groups, anaphylaxis severity or anaphylaxis triggers (p > 0.05). The median (IQR) tryptase level of the patients with idiopathic anaphylaxis was 8.25 μg/L (3.4–8.2) with a maximum level of 22.1 μg/L.

### 3.3. Triggers

**Figure 1:**
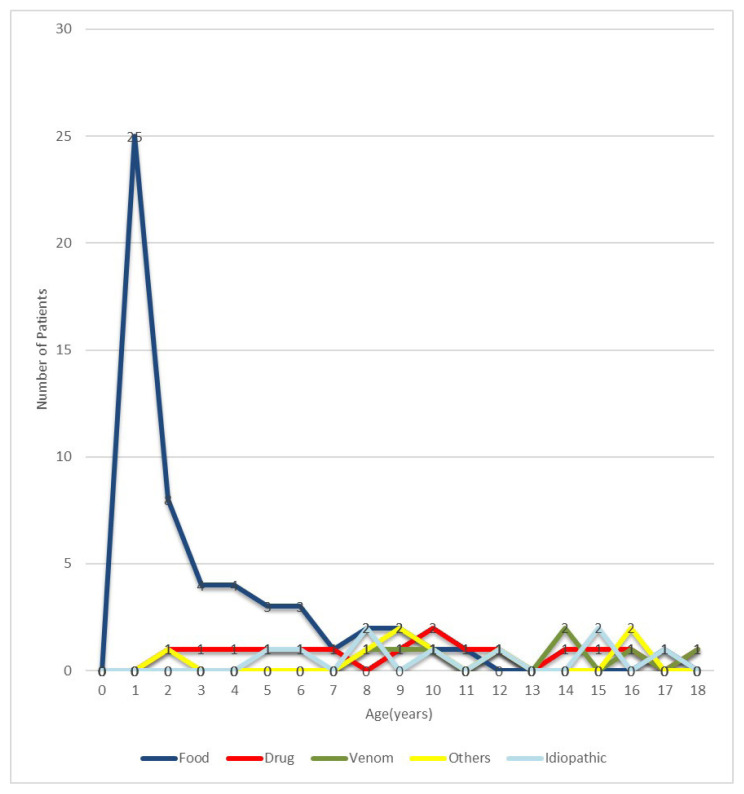
Anaphylaxis triggers according to age groups.

**Figure 2:**
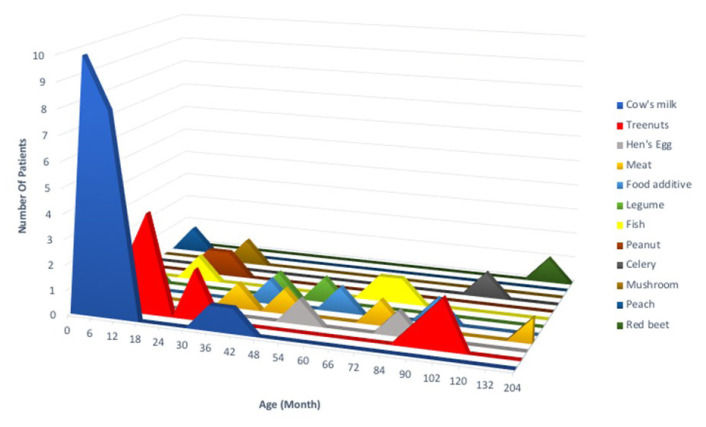
Food-related anaphylaxis triggers according to age groups.

Based on the patient’s history and diagnostic testing, nine of the cases (9.5%) were caused by an unknown allergen, eight (8.4%) by venom, fifteen (15.8%) by drugs, and the remaining (57.9%) by a food-related trigger. Cow’s milk (CM) was the most prevalent cause of food-related anaphylaxis (36.4%), followed by tree nuts (20%). Patients with food-induced anaphylaxis were younger in age (the median age at the time of anaphylaxis with foods (IQR): 13 months (7–50), the median age at the time of anaphylaxis with other than foods (IQR): 120 months (94–167), p < 0.001). The severity of food-induced anaphylaxis was milder than other triggers (p = 0.007). Cow’s milk played a significant role as a trigger in children under 2 years of age, and tree nuts as a trigger in preschoolers and school-age children. The most prevalent drug-related anaphylaxis causes were beta-lactam antibiotics (8.4%) and nonsteroidal antiinflammatory drugs (3.2%). Anaphylaxis triggers and their distribution according to age are shown in Table 3, Figures 1 and 2. There was no significant difference in sex, comorbid conditions, family history of atopy, recurrence rate, or anaphylaxis management according to anaphylaxis triggering factors (p > 0.05). There was no statistically significant difference in cutaneous findings based on different triggers (p = 0.791). However, respiratory and cardiovascular system findings were common in venom-triggered anaphylaxis (p = 0.003 and p = 0.07, respectively) and gastrointestinal system findings were more common in food-triggered anaphylaxis (p = 0.023).

### 3.4. Recurrence

Among the 144 episodes evaluated, 63 patients had a single episode, 22 had two, six had three, one had four, and three had five anaphylactic episodes with the same allergen. The most prevalent factors identified in recurring episodes were food and venom allergens. Food allergens were responsible for 22 of 32 patients who experienced recurrent anaphylaxis attacks. In two of the three patients who had five recurrent anaphylaxis episodes, the triggers were nonsteroid antiinflammatory drugs and red meat, while the attacks of the other patient were idiopathic. There was no significant difference in the recurrence rate by age group of the patients or the severity of anaphylaxis (p > 0.005).

### 3.5. Management

Information on the management of anaphylaxis was present in sixty-nine cases. Adrenaline was administered as first-line treatment by the attending health professionals intramuscularly in 39 cases (56.5%). Antihistamines (62.2%) and systemic corticosteroids (56.5%) were the most often used agents by healthcare professionals. Seventeen of 69 patients (24.6%) were treated with antihistamines alone. Inhaled SABA usage was rare (1.4%). Three patients used the adrenaline autoinjectors. A patient with peanut-induced anaphylaxis on the 2nd episode, a patient with cow’s milk-induced anaphylaxis on the 2nd episode, and a patient with idiopathic anaphylaxis on the 5th episode were treated with an adrenaline autoinjector at home by a family member.

## 4. Discussion

The current study aimed to define the characteristics of anaphylaxis according to age groups and concluded that the frequency of food-related anaphylaxis and gastrointestinal system findings in anaphylaxis episodes was much higher during infancy and decreased with increasing age. Most of the reactions in infants and young children tend to be caused by food. Food is still a common allergen in school-age children and adolescents, but more cases of anaphylactic reactions are from drugs, insect bites and unknown etiologies with increased age. The frequency of respiratory and cardiovascular system findings during anaphylaxis episodes also increased with increasing age. As well as, in older children severity of the reaction increased either. Findings reveal that children diagnosed with anaphylaxis differ in terms of etiological and clinical findings according to age groups. This difference shows the dynamically changing clinic of anaphylaxis over time and exhibits the importance of evaluating childhood anaphylaxis according to age group.

Anaphylaxis is an acute, potentially fatal systemic allergic reaction that can occur at any age with varied clinical presentations and triggers [6]. The age of anaphylaxis in our patient group varied between 2 and 214 months, with a male predominance. The youngest patient was a 2-month-old boy who had anaphylaxis with cow’s milk-based formula. A nationwide notification system of anaphylaxis including patients under 18 years of age showed that the number of male patients was higher in children [7]. The role of sex on the pathogenetic mechanisms of anaphylaxis is largely unknown however in the pediatric population, boys have a higher incidence of anaphylaxis. This is due to the higher prevalence of food allergy in males throughout this period of life, as well as the probability of increased risk-taking behaviors for anaphylaxis, particularly in adolescents. [8,9].

Food allergy was the leading cause of anaphylaxis in our study group as observed in other pediatric studies [2,3,7,10,11], being CM and tree nuts as the most common elicitor. In the first two years of life, food (most commonly CM) was almost the only anaphylaxis elicitor (32 of 34 infants), except for two infants, one of the triggering factors was a drug and the other was a vaccine. Nunes et al. [12] reported from a children’s hospital in Brazil that food was the predominant trigger and CM and nuts were the most implicated foods however in several studies CM and eggs are the most commonly reported triggers [1,13,14]. According to the network of severe allergic reactions (NORA) throughout Europe; peanuts, CM and hen’s eggs predominate as food triggers, decreasing with age [2]. South Africa is similar to Europe, with the addition of fish and tree nuts (particularly cashew nuts) [15]. In our study group, peanuts were found to be a very low-frequency trigger of anaphylaxis when compared to other studies conducted in Europe and the United States [2,16]. In particular, in our study group, we highlight the high frequency of walnut, hazelnut and cashew nuts (90.9% among tree nuts) as a suspected triggering agent, and this is probably explained by the Turkish food consumption pattern in which walnut and hazelnut are introduced early in children’s diet.^1^,^2^ In a study conducted on Portuguese children, cashew and walnut were also the most common TNs [7]. As expected, the main potential food triggers may vary in different countries depending on the food consumption pattern and lifestyles.

While food-induced anaphylaxis is usually frequent in young children, anaphylaxis caused by drugs and insect stings is often seen in older ages [17]. From infancy to puberty, there was a shift of triggers from foods to insect stings and drugs [4]. In our study group, drugs were in second place among the triggers. Initially, beta-lactam antibiotics and then nonsteroidal antiinflammatory drugs have been identified as the main cause of drug-induced anaphylaxis, in accordance with the literature [18]. The data is probably related to the high prescription rates of these drugs in childhood [19]. Venom anaphylaxis has been estimated to cause 1.5%–34% of all anaphylaxis cases [8]. The rate of venom anaphylaxis was found to be 8.4% in our study group, which is lower than the rates of venom anaphylaxis in children (20%–37%) reported from Turkey [3,11]. The low rate of venom-induced anaphylaxis in our research might be attributed to an increase in the prevalence of food allergies over time. New research may be required to update Turkey’s data on the causes of anaphylaxis.

Dhamija et al. reported a mean of 1 systemic allergic reaction per 1000 injection visits (0.1%) is estimated to have occurred in a 4-year study of subcutaneous immunotherapy (SCIT) [20]. One of our patients had urticaria, cough and wheezing after SCIT with grass pollen. Skin prick testing is a very useful diagnostic procedure for food allergies and is generally considered to be safe. Although rare, anaphylaxis may develop due to skin prick tests or prick-to-prick tests, as seen in the literature [21]. One of our patients had widespread urticaria and shortness of breath after the prick-to-prick test with fish. Latex-related anaphylaxis was observed in a patient with a history of recurrent hospitalizations and multiple surgeries due to urinary system anomaly. Depending on previous studies idiopathic anaphylaxis has been estimated to cause between 6.5% and 35% of all cases of anaphylaxis, no trigger can be identified in 9.5% of our patients [22].

Rudders et al. [23] reported that infantile anaphylaxis typically involves organ systems in the following order of frequency: skin and mucosal tissue, respiratory, and gastrointestinal systems. In our study group, cutaneous, respiratory, gastrointestinal, cardiovascular, and neurological symptoms occurred with decreasing frequency, a result consistent with findings of other studies in the literature [4]. The gastrointestinal system symptoms were more common in preschool children; however, respiratory and cardiovascular system symptoms were more common in school-age children. As similar, several studies have shown that gastrointestinal symptoms were more frequent in children at preschool age and cardiovascular system findings were less frequent in infants, and most often occurred in adolescents [7,18,24]. Nunes et al. reported more gastrointestinal symptoms than the respiratory system in infants similarly [12]. Recognition of anaphylaxis in infants can be difficult in clinical practice because infants are less verbal and the symptoms of anaphylaxis and certain normal infant behaviors may overlap. In some cases, persistent vomiting may be the only sign [24]. Vomiting was the most frequently reported symptom among gastrointestinal system findings in our study. Central nervous system findings such as behavioral changes, persistent crying, drowsiness and cessation of play are common signs of infant anaphylaxis however, the low incidence of neurologic findings found in our study could be associated with the limitation of the study methodology (retrospective design of the study). The fact that there were no recorded neurological symptoms in the infants in our study group may indicate that infant-specific neurological findings such as behavioral changes or persistent crying are not considered sufficiently in clinical practice. This also demonstrates that recognizing infantile anaphylaxis may be challenging, and the diagnostic criteria for infantile anaphylaxis should be handled separately.

In this study group, the recurrence of anaphylaxis occurred in 34% of patients. Previous pediatric studies reported a rate of anaphylaxis recurrence between 26% and 54% [4,7,25]. Although not significant statistically, foods were found to be more frequent triggers in recurrent anaphylaxis episodes. Demoly and Tanno [18] reported that fatal anaphylaxis in children is preventable because most of them are related to food accidental exposure in patients with known allergies. Three-quarters of all reactions happened at home similar to South Africa, and more frequent than reports from European Anaphylaxis Registry [4,15]. We recorded 3.2% of biphasic reactions, occurring at 4–12 h after exposure, less frequent than reports from European Anaphylaxis Registry [4].

The management of the patients diagnosed with anaphylaxis was not appropriate, as reported by other pediatric studies [12,15]. Adrenaline is the first-line treatment recommended in the guidelines but half of the patients in our study were treated with adrenaline. The rate of adrenaline use was low even in episodes of anaphylaxis that occurred in the hospital. In some studies, the underutilization of adrenaline in the treatment of anaphylaxis is more evident among infants. This is thought to be about the difficulties in identifying anaphylaxis in infants and fear of more intense adverse events [12]. However, in this study, there was no difference between infants and older children when compared to the treatment choices for anaphylaxis.

This study describes the experiences of pediatric anaphylaxis in a tertiary allergy clinic but has some limitations. The study was conducted at a single center, resulting in a limited sample size, and in a tertiary subspecialist referral setting. Retrospective evaluation of factors preceding or accompanying anaphylaxis is constrained to information recorded. Furthermore, in this study, we analyzed age-related clinical patterns of anaphylaxis, demonstrating that clinical presentation of anaphylaxis differed by age group, and these clinical distinctions were highlighted. The knowledge about dynamically changing clinics of anaphylaxis by age in the pediatric population is insufficient. Studies underlining the infantile period, in which the most significant difference is observed, have been reported in the literature recently [26]. However, in addition to the difference in the infantile period, we think that the change action continues with increasing age throughout childhood. Awareness of variable clinics in different age groups will contribute to improving the recognition and management of anaphylaxis.

In conclusion, foods come first as elicitors of anaphylaxis in preschooler children and throughout childhood. Foods are then continuously replaced by drugs, insect venom and unknown etiologies during school age. Cow’s milk and tree nuts (walnut, hazelnut and cashew nut) dominate as elicitors of food-related anaphylaxis. With increasing age, clinical manifestations of respiratory and cardiovascular system involvement and the severity of anaphylaxis increase. Although the prevalence of recurring anaphylaxis was high, the rate of adrenaline usage to treat anaphylaxis was low. Therefore, awareness of age-related symptoms in childhood anaphylaxis can aid healthcare professionals in prompt and accurate diagnosis. Improving the adequacy of anaphylaxis management is crucial both for families and healthcare professionals.

## Figures and Tables

**Table 1:** Demographic and clinical features of patients with anaphylaxis.

Patients with anaphylaxis n = 95 (%)	
**Sex** (male)	58 (61.1)
Median age at diagnosis of anaphylaxis (IQR), months	77 (29–133)
**Age distribution of the patients**	
0–24 months	34 (35.8)
24–72 months	21 (22.1)
>72 months	40 (2.1)
Presence of allergic diseases, no (%)	49 (51.6)
Atopic dermatitis	22 (23.2)
Allergic rhinitis	12 (12.6)
Asthma	11 (11.6)
Allergic proctocolitis	2 (2.1)
Urticaria- angioedema	1(1.1)
Drug allergy	1(1.1)
Family history of atopy, no (%)	29 (54.7)

**Table 2:** Demographic characteristics and symptoms of anaphylaxis according to the patients’ ages.

Age group of the patients	0–24 months n = 34 (%)	25–72 months n = 21 (%)	>72 months n = 40 (%)	*p*
**Sex**				0.815
Girl	12 (32.4)	8 (21.6)	17(45.9)	
Boy	22 (37.9)	13 (22.4)	23(39.7)	
**Presence of allergic diseases**	22 (44.9)	8 (16.3)	19 (38.8)	0.126
**Family history of atopy**	11 (34.4)	10 (31.3)	11 (34.4)	0.224
**Symptoms**				
Skin and mucosa	33 (97.1)	19 (90.5)	38 (95.0)	0.720
Respiratory	13 (38.2)	13 (61.9)	28 (70)	**0.020**
Gastro-intestinal	23 (67.6)	13 (61.9)	17 (42.5)	0.077
Cardiovascular	1 (2.9)	0	8 (20)	**0.01**4
Neurologic	0	1 (4.8)	3 (7.5)	0.269
**Anaphylaxis severity**				**0.015**
Grade 2	32(94.1)	20(95.2)	29(72.5)	
Grade 3	2(5.9)	1(4.8)	11(27.5)	
**Triggers**				
Food	32 (94.1)	16 (76.2)	7 (17.5)	**<0.001**
Drug	1(2.9)	4 (19)	10 (25)	**0.017**
Venom	-	-	8 (20)	**0.002**
Idiopathic	-	1 (4.8)	8 (20)	**0.008**
Other	1 (2.9) *	-	7 (17.5) **	**0.036**

* Other trigger of anaphylaxis is a vaccine.

** Other triggers of anaphylaxis are insect bite, subcutaneous allergen immunotherapy, latex, cold exposure and inhalant allergen.

**Table 3:** Triggering factors and location of anaphylaxis.

Triggers	Patients with food anaphylaxis n = 95 (%)
**Food**	55 (57.9)
Cow’s milk	20 (21.1)
Tree nuts	11 (11.6)
Hen’s egg	5 (5.3)
Meat	4 (4.2)
Fish	3 (3.2)
Legumes	3 (3.2)
Food additives	3 (3.2)
Peanut	2 (2.1)
Celery	1 (1.1)
Mushroom	1 (1.1)
Peach	1 (1.1)
Red beet	1 (1.1)
**Drugs**	15 (15.8)
Beta-lactam antibiotics	8 (8.4)
Nonsteroidal antiinflammatory drugs	3 (3.2)
Multiple drugs	2 (2.1)
Amikacin	1 (1.1)
Topical cream	1 (1.1)
**Idiopathic**	9 (9.5)
**Venom**	8 (8.4)
**Other**	8 (8.4)
**Location of anaphylaxis**	
House	69 (72.6)
Outdoor	14 (14.7)
Hospital	12 (12.6)

## Footnotes

1 Tarım Ürünleri Piyasa Raporu 2022 [online]. Website https://arastirma.tarimorman.gov.tr/tepge/Menu/27/Tarim-Urunleri-Piyasalari [accessed 19 November 2022].

2 Tarımsal Ekonomi ve Politika Geliştirme Enstitüsü Ürün Raporu Fındık 2021 [online]. Website https://arastirma.tarimorman.gov.tr/tepge/Menu/37/Urun-Raporlari [accessed 19 November 2022].
